# Inexpensive Mental Homes for the Middle Classes

**Published:** 1914-01-24

**Authors:** 


### Inexpensive Mental Homes for the Middle Classes.

*To the Editor of* The Hospital.

Sir,—There can be little doubt that a need has arisen for inexpensive mental hospitals for the middle classes, and that this crying need is daily becoming more insistent. Much needless suffering and discomfort are now endured by those who are unable to pay the high fees of private homes, and are therefore forced to become pauper inmates of the county mental hospitals. Hitherto, those who have friends in this position have been undesirous of calling attention to the fact through some mistaken reason—and so, much has been hidden that should undoubtedly be brought to light, for if the public knew more of the unnecessary evils to which these patients are subjected they would rise in protest, and thus draw attention to a great national want and probably much needed reform in existing institutions also. Granted there are cases in which the patient is quite incapable of appreciation of his surroundings, there are very many who are keenly alive to their position, and suffer intensely from it, from the indignities inflicted upon them by young and inexperienced warders, many of them unsuited for their responsible work, and from unnecessary rules and regulations imposed alike upon each and all regardless of personality.

For instance: from the moment a man (or woman) becomes a pauper inmate he is stripped of his belongings and attired in those provided by the authorities. These are not always clean, are ugly and ill-fitting, neither conducive to comfort or self-respect, and, in winter, are quite inadequate to maintain warmth. He is then delegated to a ward, already over-full, and left to take his chance of recovery. In some cases he may have to remain in this ward for weeks, months, or even years, varied by daily exercise in a walled enclosure, or occasional games or walks with a score or two of his companions, many of them far more afflicted than himself. Imagine the case of an educated man (or woman), accustomed to a refined, if poor, home, obliged to eat and sleep and to live in close confinement with ordinary paupers, under conditions that would arouse the ire and indignation of his saner brothers, yet, handicapped by his position, unable to protect himself (or others) or to obtain redress for his many wrongs.

Is it any wonder that so few recover in these institutions, that so many are goaded to desperation and suffer horribly, that others die of despair? Imagine the hopeless misery of those who know their state of health to have become chronic! The conditions of life in some of these institutions are far worse than those in our criminal prisons, and yet the inmates are mainly suffering from the saddest of all forms of human pain, and need personal care and individual treatment more than any other human beings, because totally deprived of freedom. There are many who would gladly and thankfully pay small weekly sums to ensure more care and comfort for their own especial cases, if suitable homes were available; this would also relieve the over-crowded county institutions, and give all more reasonable chance of recovery. Mercifully, friends are allowed to visit the patients often, and to supplement their daily rations, which, if sufficiently liberal to maintain life, are inadequate to satisfy hunger or exhaustion from which the mentally afflicted so often suffer. Surely there are some who, having experienced the benefit of kind care and wise nursing in their own hours of distress or of depression, will do what they can to help those whose sad fate is aggravated, and doubtless often increased, by misery and discomfort in a pauper lunatic asylum.

